# The KiVa antibullying program in primary schools in Chile, with and without the digital game component: study protocol for a randomized controlled trial

**DOI:** 10.1186/s13063-017-1810-1

**Published:** 2017-02-20

**Authors:** Jorge Gaete, Daniela Valenzuela, Cristian Rojas-Barahona, Eduardo Valenzuela, Ricardo Araya, Christina Salmivalli

**Affiliations:** 1 Department of Public Health and Epidemiology, Universidad de los Andes, Monseñor Alvaro del Portillo 12455, Las Condes, Santiago Chile; 2 Department of Population Health, London School of Hygiene and Tropical Medicine, Keppel Street, London, WC1E 7HT UK; 3 Faculty of Education, Pontificia Universidad Católica de Chile, Av. Vicuña Mackenna 4860, Macul, Santiago Chile; 4 Institute of Sociology, Pontificia Universidad Católica de Chile, Av. Vicuña Mackenna 4860, Macul, Santiago Chile; 5 Division of Psychology, University of Turku, Turku, FIN-20014 Finland

**Keywords:** Bullying, KiVa program, Students, Psychological functioning, School membership

## Abstract

**Background:**

Bullying is a major problem worldwide and Chile is no exception. Bullying is defined as a systematic aggressive behavior against a victim who cannot defend him or herself. Victims suffer social isolation and psychological maladjustment, while bullies have a higher risk for conduct problems and substance use disorders. These problems appear to last over time. The KiVa antibullying program has been evaluated in Finland and other European countries, showing preventive effects on victimization and self-reported bullying. The aims of this study are (1) to develop a culturally appropriate version of the KiVa material and (2) to test the effectiveness of the KiVa program, with and without the online game, on reducing experiences of victimization and bullying behavior among vulnerable primary schools in Santiago (Chile), using a cluster randomized controlled trial (RCT) design with three arms: (1) full KiVa program group, (2) partial KiVa (without online game) program group and (3) control group.

**Methods and design:**

This is a three-arm, single-blind, cluster randomized controlled trial (RCT) with a target enrolment of 1495 4th and 5th graders attending 13 vulnerable schools per arm. Students in the full and partial KiVa groups will receive universal actions: ten 2-h lessons delivered by trained teachers during 1 year; they will be exposed to posters encouraging them to support victims and behave constructively when witnessing bullying; and a person designated by the school authorities will be present in all school breaks and lunchtimes using a visible KiVa vest to remind everybody that they are in a KiVa school. KiVa schools also will have indicated actions, which consist of a set of discussion groups with the victims and with the bullies, with proper follow-up. Only full KiVa schools will also receive an online game which has the aim to raise awareness of the role of the group in bullying, increase empathy and promote strategies to support victimized peers. Self-reported victimization, bullying others and peer-reported bullying actions, psychological and academic functioning, and sense of school membership will be measured at baseline and 12 months after randomization.

**Discussion:**

This is the first cluster RCT of the KiVa antibullying program in Latin America.

**Trial registration:**

ClinicalTrials.gov, Identifier: NCT02898324. Registered on 8 September 2016.

**Electronic supplementary material:**

The online version of this article (doi:10.1186/s13063-017-1810-1) contains supplementary material, which is available to authorized users.

## Background

All children and youth should be offered a safe learning environment where they are treated equally and with respect. Research on school bullying has shown that despite many efforts, this ideal is far from reality for many school-aged children. Bullying, defined as repeated aggressive or harmful actions directed at a less powerful peer [1, 2], is highly prevalent in schools worldwide. In a large study involving 40 countries, 10.7% of students reported bullying others on a regular basis, 12.6% said that they were repeatedly bullied by their peers, and 3.6% reported being both perpetrators and victims of bullying [3]. Large-scale surveys conducted in Chile, although scarce, have indicated similar or even higher prevalence of the problem [4, 5].

Bullying compromises equal learning opportunities, safety and wellbeing of millions of children and youth around the world. Research shows that the quality of peer relations, especially emotional support (or lack of it) from peers, has significant consequences for school motivation, engagement and achievement [6]. Apart from reducing targeted students’ liking of (and presence at) school, as well as their motivation and achievement, victimization has short- and long-term psychosocial and mental health consequences, such as depression and anxiety [7, 8]. Bullying perpetration, on the other hand, is associated with later criminal offending [9]. Besides victims and perpetrators, bullying even has negative outcomes for classmates who are merely witnessing the bullying [10, 11].

The call for effective prevention of bullying has resulted in numerous school-based programs developed for this purpose. According to the meta-analysis by Ttofi and Farrington [8], such programs reduce the prevalence of bullies and victims on average by 17–23% but the effects of different programs vary substantially. Furthermore, even programs that were proven to be effective in one study, often in their country of origin, have sometimes produced little or no effects in replication studies in new contexts. More research on the generalizability of evidence-based programs across (culturally) diverse groups, countries and contexts is needed. Carrying out such research in Chile, where there is urgent need for evidence-based prevention of bullying, is highly relevant. Despite government-supported initiatives and guidelines to help schools deal with bullying (“Ley N° 20536: Sobre violencia escolar,” 2011 [12]), no randomized controlled trials (RCT) have been carried out in Chile in order to test the effectiveness of existing antibullying programs.

The KiVa antibullying program (an acronym for Kiusaamista Vastaan, “against bullying”), developed at the University of Turku, is widely used in Finnish schools. KiVa has been evaluated in a large RCT and during its nationwide dissemination in Finland [13–15], with positive effects being shown on numerous variables, including bullying and victimization, depression, anxiety, enjoyment of school and academic motivation. There are also experiences in other parts of Europe showing good results [16, 17]. The KiVa program involves indicated actions (targeted at students who have been involved in bullying as perpetrators or victims): they refer to a series of discussions with the bullies and their victims with a proper follow-up procedure. The universal actions (targeted at all students as potential witnesses of bullying) on the other hand, consist of ten 2-h student lessons delivered during a school year, with the aim to raise awareness of the role of the peer group in bullying, increase empathy towards bullied students, and promote bystander strategies to support and defend their victimized peers. The universal actions also include posters, highly visible vests for recess supervisors and, finally, an innovative digital learning environment, an antibullying computer game [18] that can be played with PC or tablet computers, either during (there are special slots deserved for this in the curriculum) or between lessons.

The digital KiVa game involves five levels, the topics of which match the contents of the 10 student lessons. Each level includes three modules: “I Know,” “I Can,” and “I Do.” In the I Know module, the students learn new facts about bullying, but also test what they have learnt during the lessons so far. They are asked questions about the contents of the lessons in game-like quizzes, and they can test themselves with respect to different characteristics of bullying situations (e.g., Can I resist group pressure? What are my best ways to support a victimized peer?). In I Can module, the students practice the skills that they have learnt during the student lessons. They move around in a virtual school and face challenging situations in the playground, lunchroom and school corridors. They make decisions regarding how to respond to these situations, and get feedback based on their choices. In certain points of the narration the player has an opportunity to “read the minds” of the other characters (i.e., the victim or the bystanders), seeing how they think and feel. Based on these cues, and on how the episode proceeds, the player can also change their behavior and try out something different. The third module, I Do, is designed to encourage the students to make use of their acquired knowledge and skills in real-life situations. This happens by asking them to report – at each level of the game – which ones of the KiVa rules (that were adopted during the lessons) they have put into practice; for instance, whether they have treated others with respect, whether they have resisted negative group pressure, or whether they have supported someone who has been bullied. Finally, they get feedback of their performance.

A challenge of KiVa, as well as other multicomponent prevention/intervention programs, is that the relative effectiveness of the different components is unknown. Such knowledge is much needed to inform further development of the programs and to adapt them into new contexts. The implementation of the digital game as part of the KiVa program, for instance, should be justified by sound evidence showing that stronger effects can be obtained when the online game is implemented together with the other components of the program. From the public health perspective, it is necessary to assess interventions regarding their cost-effectiveness in order to provide to the community an effective intervention with the lowest cost.

In this study, investigators from Chile and Finland will collaborate on adapting the KiVa antibullying program for schools in Chile and evaluating its effectiveness in this new context. We will further investigate whether the digital game included in the KiVa program adds to the effects obtained when the program is implemented without this component. Besides its huge practical significance, the project will contribute to the scientific discussion on the replicability of antibullying interventions in new contexts, and on the possibilities of new Information and Communication Technologies (ICT) tools to reduce bullying.

## Methods and design

This is a three-arm, single-blinded (blinded only to the outcome evaluator), cluster RCT, which will compare the effectiveness of the KiVa antibullying program with the digital game component (full KiVa schools) versus the KiVa program without the digital game component (partial KiVa schools) versus usual management for bullying (control schools) in low-income schools in Santiago, Chile. Methods are in accordance with the Standard Protocol Items: Recommendations for Interventional Trials (SPIRIT) Statement (Additional file 1).

### Aims and hypothesis

#### General aim

To develop a culturally appropriate version of the KiVa antibullying program (specifically, unit 2 which is targeted at 10–12-year-old children) and to carry out a cluster RCT to test its effectiveness with and without the digital game component versus usual management for bullying in students attending years 5 and 6 in low-income schools in Santiago, Chile. The project involves two stages: first, formative work, where the research teams translate, review and adapt the KiVa program to Chile; and second, the cluster RCT.

#### Specific aims: formative work

1. To translate and adapt all the material (unit 2 of the KiVa program and the evaluation tools): the translation will be done by professional translators and reviewed by young researchers, supervised by the principal investigators (PI) and coinvestigators. Necessary adaptations – mainly to the surface structure of the program – will be done during the process
2. To validate and measure the psychometric properties of the Revised Olweus Bully/Victim Questionnaire (OBVQ): a cross-sectional study will be carried out to assess the validity and reliability of the questionnaire in a sample of students of the same age and background as the participants in the RCT. Along with the revised OBVQ, the students will answer a questionnaire that has been used to assess bullying in Chile, the Survey of Maltreatment and Abuse of Power among Students (MIAP) [19], in order to test concurrent validity

#### Specific aims: cluster randomized controlled trial

1. To compare the level of self-reported victimization of 5th and 6th graders attending full KiVa, partial KiVa and control in low-income schools
2. To compare the level of self-reported bullying actions of 5th and 6th graders attending full KiVa, partial KiVa and control in low-income schools
3. To compare the level of peer-reported victimization of 5th and 6th graders attending full KiVa, partial KiVa and control in low-income schools
4. To compare the level of peer-reported bullying actions of 5th and 6th graders attending full KiVa, partial KiVa and control in low-income schools
5. To compare the level of self-reported psychological difficulties of 5th and 6th graders attending full KiVa, partial KiVa and control in low-income schools
6. To compare the level of self-reported psychological strengths of 5th and 6th graders attending full KiVa, partial KiVa and control in low-income schools

### Hypothesis

We hypothesize that at the end of the intervention there will be fewer students identified as targets and perpetrators of bullying in schools receiving the KiVa intervention than in control schools. Furthermore, we expect that the program effects will be stronger when the online game is implemented together with the other program components. The latter hypothesis is based on the emerging scientific literature on the usefulness of ICT tools in interventions for children and youth [20]. Finally, we hypothesize that the psychosocial adjustment of students will increase more in schools where bullying problems decrease the most.

### Setting and population

The Chilean educational system is structured into three types of primary and secondary schools: municipal state-funded schools (40.38%), subsidized schools (administered by private organizations but also receiving state funds, 52.13%) and private schools (administered by private organizations, not receiving state funds, 7.50%). The quality of the education received by students in municipal state-funded schools, based on standardized tests such as PISA [21], is lower than in private and subsidized schools. There are also more bullying problems in state-funded schools, as compared with the other types of schools (17.2% in state schools, 13% in subsidized schools, 10% in private schools) [5]. Our target sample will be these most vulnerable schools in Santiago, with students mainly coming from low- to middle-income families.

### Inclusion criteria

Our sample frame comprised all schools having primary education (year 1 to year 8), mixed-sex, located in Santiago, with two or three classes per year level and having a vulnerability index (School Vulnerability Index – National System of Equality Allocation (IVE-SINAE)) ≥75%. The IVE-SINAE is built taking into account several students’ and parental variables: health, family income, receiving state benefits. This percentage means the proportion of students in a school who are in most need.

All eligible schools will be invited to participate.

### Recruitment/allocation of schools

**Fig. 1:**
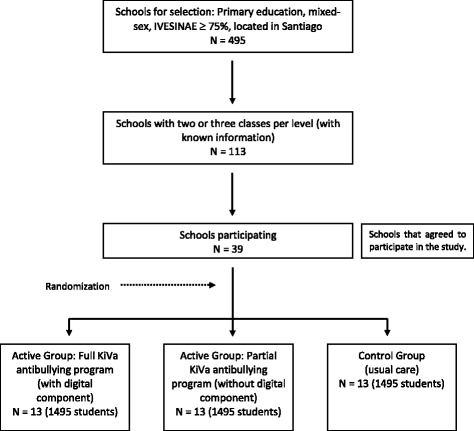
Flowchart

Randomization will be performed once all schools are recruited and after baseline in order to obtain balance with respect to size of the schools and level of self-reported victimization. Schools will be randomly assigned to either group with a 1:1:1 allocation as per a computer-generated randomization. An independent statistician will perform the randomization and allocation concealment will be ensured informing the allocation until all students have been recruited into the trial, which takes place after all baseline measurements have been completed (see Fig. 1).

### Recruitment of students and consent process

After the school authorities accept to participate in the study, students and their parents will be informed of the study. Parents will be informed that the KiVa program will be part of the school curriculum following approval by the school and educational authorities. Also, the team will send an Informed Consent Form to parents to provide an opportunity to request the withdrawal of their children from the study assessments. Baseline information will be collected from all students, but those whose parents express their option for a withdrawal will not be subsequently assessed.

### Implementing the KiVa program

The intervention will be implemented during the 2017 academic year, running from March to November. All schools in Chile have one academic hour per week free of teaching and homework, normally used to promote nonacademic abilities among students. This period of time, already in the curriculum, will be used to deliver the KiVa program.

Universal actions consist of (1) ten 2-h monthly student lessons delivered by a trained teacher and a facilitator (member of the research team), (2) posters encouraging students to support victims and behave constructively when witnessing bullying, (3) a member of the school designated by the school authorities will be present in all school breaks and at lunchtime using a visible KiVa vest to remind everybody that they are in a KiVa school, (4) parents will be informed about bullying through a website, letters and meetings. Finally, in schools receiving the KiVa program with the digital learning environment there will be (5) a tablet-based online game, used by the students during and between their lessons. Playing the game, the students will experience bullying situations in a virtual environment where they will have the opportunity to practice the skills learned in the student lessons to deal constructively with these situations.

The KiVa program also includes “indicated actions.” Bullying incidents will be managed by a designated KiVa team in the school (a teacher, a psychologist or any other professional designated by school authorities). These people will organize discussions with the students involved in bullying and will encourage prosocial peers of the victimized students to support them.

The research teams will provide all the material, training and coaching during the study. Certified KiVa trainers (JG and DV) will deliver the training to all teachers and involved school staff during 2 days in the beginning of the academic year. The Chilean team, backed up by the Finnish team, will be coaching and supporting the intervention schools throughout the academic year. The Chilean team will also undertake observations of how the universal and indicated actions are delivered, in order to assure the fidelity to the program.

The school allocated in the partial KiVa program will receive all actions with the exception of the digital game.

### Control arm

The control group will receive the normal teaching activities and, if the study shows that the intervention is effective, they will receive the intervention in the following year.

### Outcome measures

As demographic information, data on child sex and date of birth, family conditions and socioeconomic features will be collected from children and parents. Permission will be asked to have access to the registry of the academic performance as the grade point average (GPA) of the participating students each trimester.

#### Primary outcomes measure

The primary outcome variables, bullying and victimization, will be measured by the 40-item Revised Olweus Bully/Victim Questionnaire [1], identifying self-reported victims, bullies and bully-victims. The questionnaire has been used worldwide to measure the prevalence of bullying and victimization [22, 23], and the effectiveness of antibullying programs [15, 24, 25]. The questionnaire will be translated into Spanish and its psychometric properties will be evaluated during the formative work phase in a cross-sectional survey among 4th to 8th graders in a sample with similar background and vulnerabilities to the one that will participate in the RCT.

#### Secondary outcome measure

Psychosocial adjustment of students will be assessed with the Strengths and Difficulties Questionnaire (SDQ). This questionnaire assesses 25 attributes into five subscales: (1) Emotional symptoms (five items), (2) Conduct problems (five items), (3) Hyperactivity/inattention (five items), (4) peer relationship problems (five items) and prosocial behavior (five items). The first four subscales generate a total score of difficulties (20 items) and the prosocial behavior scale is considered to reflect the personal strengths of individuals. There are versions for parents and teachers of children aged 4 to 16 years [26] and a self-reported questionnaire for children aged 11 to 16 years [27]. The self-reported version has also been used in younger children (aged 8–13 years) with satisfactory results [28]. It has been widely used [29–36] and has demonstrated good psychometric properties [37]. The authors have the permission to use the Spanish version of SDQ and they have already surveyed around 600 parents and children (aged 9 to 15 years) attending state schools, with similar background and vulnerabilities to the schools expected to participate in the RCT, to carry out a study of the validity and reliability of this questionnaire. The parent and child versions of the SDQ will be utilized.

#### Other outcomes

The Psychological Sense of School Membership (PSSM) scale is a self-reported instrument developed to assess the sense of school belonging. The original PSSM scale comprises 18 items: 13 positively worded statements and 5 negatively worded statements. For each statement, students answer on a 5-point scale (1 = not at all true; 5 = completely true). All of these items are related to students’ perceptions of being “accepted, respected, included and supported by others in the school social environment” (p. 80) [38]. It has been widely used – mainly in English-speaking countries – and it has been associated with several variables related to academic achievement such as increased competence and self-efficacy [39], increased school attendance [40] and higher grades [41]. The authors have conducted a validation study with a sample of 1250 early adolescents in Chile [42]. Both exploratory and confirmatory factor analyses provide evidence of an excellent fit for a one-factor solution after removing the negatively worded items. The new 13-item scale has an internal consistency of 0.92. We also have conducted preliminary analyses finding that high school membership was associated with better academic performance, stronger school bonding, a reduced likelihood of school misbehavior and reduced likelihood of substance use. Analyses showed support for the reliability and validity of the PSSM among Chilean adolescents.

### Sample size

To obtain a significant mean difference between groups, we expect to recruit 39 schools allocated on a 1:1:1 ratio. Each arm should include 1495 eligible students. We used the results in a previous study [15] and the clustersampsi command in Stata Software estimating the number of clusters in two arms (full Kiva versus control; and partial KiVa versus control), using the following command:

clustersampsi, samplesize mu1(*X1*) mu2(*X*2) sd1(*Y1*) sd2(*Y27*) m(*Z*) rho(*R*)

Where *X1* = mean in arm 1; *X*2 = mean in arm 2; *Y1* = standard deviation (SD) in arm 1; *Y2* = SD in arm 2; *Z* = number of children per school on average (harmonic mean) (*n* = 115); and *R* = intracluster correlation

The three arms will be balanced with respect to school size. All students attending 4th and 5th grade in 2016 at selected schools will participate in the study.

### Data collection

The preintervention assessment will be carried out at the end of the academic year in November (2016), when participants are attending year 4 and year 5, and the post-intervention assessment 1 year later (November 2017) when students are attending year 5 and year 6, respectively. Independent researchers blind to the allocation will supervise the assessments in classrooms; they will receive a full day of training to ensure a fully standardized data collection. Students will fill self-reported questionnaires with the OBVQ, SDQ and PSSM measures during regular school hours. See Table 1 for the explanatory SPIRIT diagram.

**Table 1:**
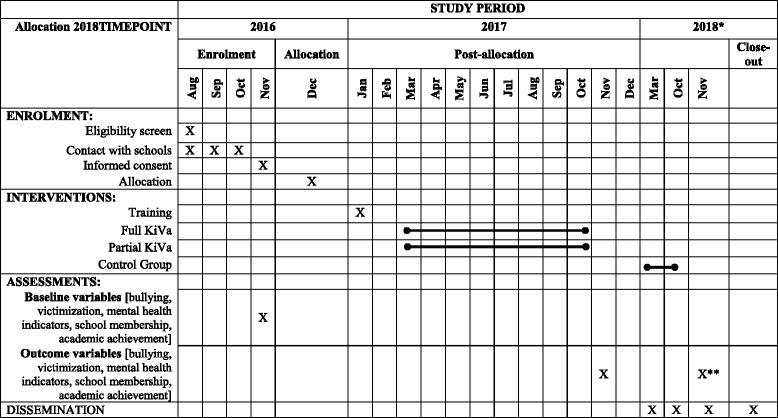
Standard Protocol Items: Recommendations for Interventional Trials (SPIRIT) diagram

^*^If any of the KiVa groups is more effective than the control group, schools in the control group will receive the KiVa program during 2018. ^**^Assessment in control-group schools only

### Data management

After the participants have completed the questionnaires, the data will be entered into a secure platform, without identifying information (each participant will be assigned an ID number). The original copies of the instruments will be filed and stored, under lock and key, in the PI’s office, along with the list linking the participants’ names and ID numbers. Only two research assistants, in charge of data entry, and the statistician will have access to the database.

### Data analyses

The data analyses will follow Consolidated Standards of Reporting Trials (CONSORT) guidelines for RCTs [43]. General school features (size, number of teachers, etc.) will be used to compare participating schools with the ones that were invited but did not participate. Descriptive statistics will be used to compare the three arms at baseline. Primary comparative analysis will be conducted on an intention-to-treat basis. We will use multivariable regression to study differences in mean bullying behavior scores (primary outcome) between groups at the end of the intervention, controlling for baseline outcome variable scores and taking into account clustering within classes and schools. Secondary analysis will be conducted considering adjustment for baseline scores, age and sex. Psychosocial adjustment, psychological sense of school membership and academic outcomes will be analyzed with the same approach.

Sensitivity analysis making different assumptions will be conducted to investigate the potential effects of missing data. Multiple imputation will be performed if necessary.

Apart from evaluating the effects of KiVa, the data will enable various other analyses concerning factors associated with bullying problems and at the individual, classroom and school level in the Chilean context.

### Trial management

The study will comply with local Research Governance requirements.

## Discussion

The proposed study is the first to test the effectiveness of a school-based antibullying program in Chile in a RCT, and the first study evaluating the KiVa program in its Spanish version. Additionally, testing the added value of the digital learning environment will provide important information on whether the online game should be included in case of wide dissemination of the program in Chile.

A model for disseminating the KiVa program outside Finland already exists, and has been implemented successfully in several European countries. Thus, if the program effects are positive, wide implementation in Chile and other Latin American countries is possible in the near future.

There are, however, some potential risks. An obvious threat for the validity of the findings is that the implementation quality remains low in schools that will be recruited into the RCT. To avoid this threat, we will arrange face-to-face training sessions with the school personnel in order to motivate them to implement the KiVa program as intended, as well as ongoing support and coaching during the implementation process.

Another risk might be difficulty in recruiting enough schools into the RCT. To minimize this risk, we will prepare the recruitment carefully and inform the schools in good time, utilizing the excellent networks of the members of the Chilean team.

### Trial status

This study will start recruiting participants in November 2016.

## Additional files

Additional file 1: SPIRIT 2013 Checklist: recommended items to address in a clinical trial protocol and related documents. (DOC 122 kb)
Additional file 2: Original Funding Document in Spanish. (PDF 966 KB)
Additional file 3: English translation of Funding Document. (PDF 155 KB)
Additional file 4: Funding Document from the Academy of Finland. (PDF 48.3 KB)
Additional file 5: Original Ethical Approval in Spanish. (PDF 63.3 KB)
Additional file 6: English translation of Ethical Approval. (PDF 195 KB)

## Abbreviations

CONSORT: Consolidated Standards of Reporting Trials
IVE-SINAE: School Vulnerability Index – National System of Equality Allocation [Índice de Vulnerabilidad Escolar-Sistema Nacional de Asignacion con Equidad]
KiVa: Kiusaamista Vastaan, “against bullying”
MIAP: Survey of Maltreatment and Abuse of Power among Students
OBVQ: Revised Olweus Bully/Victim Questionnaire
PSSM: Psychological Sense of School Membership scale
RCT: Randomized controlled trial
SD: Standard deviation
SDQ: Strengths and Difficulties Questionnaire
