# Supplementary material for: The KiVa antibullying program in primary schools in Chile, with and without the digital game component: study protocol for a randomized controlled trial
Source: Trials. 2017 Feb 20;18:75. doi: 10.1186/s13063-017-1810-1 (PMC5319041; doi:10.1186/s13063-017-1810-1)
Supplement: Additional file 2: — Original Funding Document in Spanish. (PDF 966 KB) [file 13063_2017_1810_MOESM2_ESM.pdf]

CONICYT
Ministerio de Educación
Gobierno de Chile

DOCUMENTO ELECTRONICO

APRUEBA ADJUDICACIÓN DE PROYECTOS DE INVESTIGACIÓN CONJUNTA CHILE-FINLANDIA EN TECNOLOGÍAS Y NUEVOS ENTORNOS DE APRENDIZAJE, CONVOCATORIA 2015, PROGRAMA DE COOPERACIÓN INTERNACIONAL DE CONICYT.
RESOLUCION EXENTO Nº: 1978/2016
Santiago15/02/2016

VISTOS:
Lo dispuesto en el DS Nº491/71; DS N°97/15, ambos del Ministerio de Educación; Ley de Presupuestos del Sector Público Nº 20.882 para el año 2016; DS 97/2015 del Ministerio de Educación y Resolución Nº 1600 de 2008, de Contraloría General de la República, y

CONSIDERANDO:

a. La Resolución Exenta Nº533/2015 de CONICYT, que aprobó las bases de postulación para Proyectos de Investigación Conjunta Chile-Finlandia en Tecnologías y Nuevos Entornos de Aprendizaje, Convocatoria 2015, Programa de Cooperación Internacional de CONICYT.

b. Memorando TED N°969/2016 del Programa de Cooperación Internacional de CONICYT, que solicita emitir la Resolución de Fallo para el Concurso AKA-CONICYT en Ciencias de la Educación, Proyectos de Investigación Conjunta Chile-Finlandia en Tecnologías y Nuevos Entornos de Aprendizaje, Convocatoria 2015, y adjunta antecedentes.

c. La Resolución Exenta N°8018/2015 de CONICYT, que aprobó la Constitución del Comité de Área del presente concurso.

d. La convocatoria del certamen aludido, publicada en el Diario El Mercurio de Santiago, de fecha 29 de mayo de 2015.

e. Listado total de proyectos presentados al Concurso AKA-CONICYT en Ciencias de la Educación.

f. Proyecto declarado fuera de bases, por no contar con contraparte de Finlandia.

g. Proyectos no seleccionados del Concurso AKA-CONICYT en Ciencias de la Educación (Anexo 2)

h. Listado de proyectos seleccionados para adjudicación (4).

i. Acta de selección Concurso proyectos de investigación conjunta CONICYT Chile-Academia de Finlandia en Ciencias de la Educación-Convocatoria 2015, de fecha 05 de noviembre de 2015.

j. Acta de Selección Final CONICYT/AKA-llamado conjunto en Tecnologías y Nuevos Entornos de Aprendizaje, de diciembre de 2015.

k. Cuadro presupuesto de los adjudicados por año.

l. Certificado de disponibilidad presupuestaria N°07 de 13 de enero de 2016, del Departamento de Administración y Finanzas de CONICYT, que indica que se cuenta con la disponibilidad presupuestaria con la cual se podrá financiar el compromiso que se tramita en el documento.

m. Las facultades que detenta esta Dirección Ejecutiva, de conformidad a lo señalado en el DS 491/71, el DS 97/15, ambos del Ministerio de Educación.

RESUELVO:

1. APRUÉBASE la adjudicación del CONCURSO DE PROYECTOS DE INVESTIGACIÓN CONJUNTA CHILE-FINLANDIA EN TECNOLOGÍAS Y NUEVOS ENTORNOS DE APRENDIZAJE, CONVOCATORIA 2015, de conformidad a lo dispuesto en Acta de Selección Final CONICYT-AKA, del llamado conjunto en Tecnologías y Nuevos Entornos de Aprendizaje, de diciembre de 2015, que incluye a los siguientes proyectos:

Ch.Code	Title	PI Chile	Ch Institution	PI Finland	Fin. Institution	Other Researchers Ch.	Aporte CONICYT (pesos chilenos)
AKA-EDU/ 15	KiVa anti-bullying program in Chile: Evaluation of effectiveness with and without the digital game component	Jorge Gaete	Universidad de los Andes	Christina Salmivalli	University of Turku	Christian A. Rojas-Barahona; Professor Eduardo Valenzuela (OUC)	$ 149.963.000
AKA-EDU/ 11	Engaging Learning Practices in STEM: Research collaboration with Finland and Chile	Beatriz Ávalos	CIAE U. de Chile	Jari M J Lavonen	University of Helsinki,	Patricio Cumsille; Valeria M. Cabello; xxxUribe (PUC)	$ 150.000.000
AKA-EDU/ 03	ENHANCING LEARNING AND TEACHING FOR FUTURE COMPETENCES OF ONLINE INQUIRY IN MULTIPLE DOMAINS	Mario	PUC	Maria Vauras	Faculty of Education, University of	Roberto González-Ibáñez	$ 150.000.000

AKA-EDU/ 03	(IFUCO)	Quintanilla	PUC	Marja Vauras	Turku	(USACH)	$ 150.000.000
AKA-EDU/ 01	Learning environments for STEM	Roberto Araya	Centro de Investigación Avanzada en Educación (CIAE), University of Chile	Jouni Viiri	Department of Teacher Education, University of Jyväskylä (JYU)	Jorge Soto-Andrade; Raúl Gormaz;	$ 150.000.000
**Total**							**$ 599.963.000**

2. NOTIFÍQUESE por el Programa de Cooperación Internacional el resultado del certamen a todos(as) los(as) investigadores(as) responsables de los proyectos postulados en Chile y celébrense los respectivos convenios con los adjudicados.

3. El Departamento de Administración y Finanzas imputará el gasto que irrogue la presente resolución, a la cuenta presupuestaria que corresponda.

4. ANÓTESE por el Oficial de Partes el número y fecha de la presente resolución, que complementa, en el campo "DESCRIPCIÓN" ubicado en el Repositorio de Archivo Institucional, en el documento digital de la Resolución Exenta N°533/2015 de CONICYT.

5. REMÍTASE copia de la presente resolución a Presidencia, al Programa de Cooperación Internacional, al Departamento de Administración y Finanzas, al Departamento Jurídico y a la Oficina de Partes.

6. DÉJASE constancia que contra el presente acto administrativo procede el recurso de reposición de conformidad a lo previsto en la Ley N° 19.880, que establece las Bases de los Procedimientos Administrativos que rigen los Actos de los Órganos de la Administración del Estado, sin perjuicio de otros medios de impugnación contemplados por el ordenamiento jurídico.

ANÓTESE, REFRÉNDESE, COMUNÍQUESE Y NOTIFÍQUESE

UNIDAD DE PRESUPUESTO - CONTABILIDAD	
FECHA	11-02-2016
ITEM	24.01.223
CENTRO DE COSTO	COOPERACION INTERNACIONAL
ANALISTA	Alejandra Moraga Vásquez

CHRISTIAN NICOLAI ORELLANA
Director(a) Ejecutivo
DIRECCION EJECUTIVA

CNO / / MMF / SQG / mvc

DISTRIBUCION:
CATALINA PALMA - Coordinador(a) de Coop. Inter. en Investigación Conjunta - RELACIONES INTERNACIONALES
RICARDO CONTADOR - Coordinador(a) de Finanzas Unidad de Coop. Internacional - RELACIONES INTERNACIONALES
INGRID MARLENE TAPIA - Secretaria Dirección - RELACIONES INTERNACIONALES
RODRIGO MONSALVE - Director(S) - RELACIONES INTERNACIONALES
OFICINA DE - Buzón Oficina de Partes - GESTION DE PERSONAS

Firmado Electrónicamente en Conformidad con el Artículo 2º letra F y G de la Ley 19.799
