# Supplementary material for: The KiVa antibullying program in primary schools in Chile, with and without the digital game component: study protocol for a randomized controlled trial
Source: Trials. 2017 Feb 20;18:75. doi: 10.1186/s13063-017-1810-1 (PMC5319041; doi:10.1186/s13063-017-1810-1)
Supplement: Additional file 3: — English translation of Funding Document. (PDF 155 KB) [file 13063_2017_1810_MOESM3_ESM.pdf]

SGDOC

APPROVES AWARD OF JOINT RESEARCH PROJECTS CHILE-FINLAND IN TECHNOLOGIES AND NEW LEARNING ENVIRONMENTS, CALL 2015 INTERNATIONAL COOPERATION PROGRAM OF CONICYT.
EXEMPT RESOLUTION Nº 1978/2016
Santiago 15/02/2016

ELECTRONIC DOCUMENT

SEEN:
The provisions of the DS Nº 491/71; DS 97/15, both of the Ministry of Education; Budget Law Nº 20.882 Public Sector 2016; DS 97/2015 of the Ministry of Education and Resolution Nº 1600 of 2008, General Comptroller of the Republic, and

CONSIDERING:

a. Exempt Resolution Nº 533/2015 of CONICYT, which approved the nomination bases for Joint Research Projects Chile-Finland in Technologies and New Learning Environments, Call 2015, International Cooperation Program of CONICYT.

b. TED Memorandum Nº 969/2016 International Cooperation Program of CONICYT, requesting issue the resolution decision for AKA-CONICYT Contest in Education Sciences, Joint Research Projects Chile-Finland Technology and New Learning Environments, Call 2015 and attached background.

c. Exempt Resolution Nº 8018/2015 of CONICYT, which approved the Constitution Area Committee of the present contest.

d. The Call of the aforementioned contest, published in the newspaper El Mercurio of Santiago, dated May 29, 2015.

e. List of projects submitted to the AKA-CONICYT Contest in Education Sciences.

f. Project declared out of bases, for not having a Finnish counterpart.

g. Projects not selected in the AKA-CONICYT Contest in Education Sciences (Annex 2).

h. List of selected projects for adjucation (4).

i. Selection Contest Act of Joint Research Projects CONICYT Chile-Academy of Finland in Education Sciences-Call 2015, dated November 5th, 2015.

j. Final Selection Act CONICYT/AKA-called set in Technology and New Learning Environments, December 2015.

k. Table Budget allocated per year.

l. Budget availability certificate Nº 07 of January 13th, 2016, the Department of Administration and Finance of CONICYT, indicating that budgetary availability features with which it may finance the commitment that is processed in the document.

m. The faculties that holds the Executive Board, in accordance with which is stated in DS 491/71, the DS 97/15, both of the Ministry of Education.

RESOLVE:

1. To approve the adjudication of the CONTEST JOINT RESEARCH CHILE-FINLAND IN TECHNOLOGIES AND NEW LEARNING ENVIRONMENTS, CALL 2015 in accordance with the provisions of Final Act Selection CONICYT-AKA, of the joint call in Technologies and New Learning Environments, of December 2015, which includes the following projects:

Ch.Code	Title	PI Chile	Ch Institution	PI Finland	Fin. Institution	Other Researchers Ch.	Aporte CONICYT (pesos chilenos)
AKA-EDU/ 15 (OUC)	KiVa anti-bullying program in Chile: Evaluation of effectiveness with and without the digital game component	Jorge Gaete	Universidad de los Andes	Christina Salmivalli	University of Turku	Christian A. Rojas-Barahona; Professor Eduardo Valenzuela	$ 149.963.000
AKA-EDU/ 11	Engaging Learning Practices in STEM: Research collaboration with Finland and Chile	Beatriz Avalos	CIAE U. de Chile	Jari M J Lavonen	University of Helsinki	Patricio Cumsille; Valeria M.	$ 150.000.000
AKA-EDU/ 03	ENHANCING LEARNING AND TEACHING FOR FUTURE COMPETENCES OF ONLINE INQUIRY IN MULTIPLE DOMAINS	Mario Quintanilla	PUC	Marja Vauras	Faculty of Education, University of Turku	Roberto Gonzalez-Ibañez (USACH)	$ 150.000.000
AKA-EDU/ 01	Learning environments for STEM	Roberto Araya	Centro de Investigacion Avanzada en Educacion (CIAE), Universidad de Chile	Jouni Viiri	Department of Teacher Education, University of Jyvaskyla (JYU)	Jorge Soto-Andrade; Raul Gormaz	$ 150.000.000
Total							$599.963.000

2. NOTIFY by the International Cooperation Program the result of the contest to all researchers responsible for projects in Chile and should be celebrated the respective agreements adjudicated.

3. The Department of Administration and Finance impute expenditure incurred to this resolution, to the appropriate budget account.

4. REGISTER by the Parties Officer the number and date of this resolution, supplementing the "DESCRIPTION" field located in the Institutional Repository File in the digital document Exempt Resolution Nº 533/2015 of CONICYT.

5. REFER copy of this resolution to the President, the Program for International Cooperation, the Department of Administration and Finance, the Legal Departament and the Office of Parties.

6. LEAVE record against this administrative act the remedy of reinstatement in accordance with the provisions of Law Nº 19.880, which establishes the basis of the Administrative Procedures governing the Acts of the Management Bodies of the State Administration, without prejudice to other means contesting covered by the law.

WRITE, COUNTERSIGN, COMMUNICATE AND NOTIFY

BUDGET UNIT – ACCOUNTING	
DATE	11-02-2016
ITEM	24.01.223
COST CENTER	INTERNATIONAL COOPERATION
ANALYST	Alejandra Moraga Vásquez

CHRISTIAN NICOLAI ORELLANA
Executive Director
EXECUTIVE MANAGEMENT

CON/MMF/SQG/mvc

DISTRIBUTION:
CATALINA PALMA – Coordinator of International Cooperation in Joint Research – INTERNATIONAL RELATIONS
RICARDO CONTADOR – Coordinator of the International Cooperation Finance Unit – INTERNATIONAL RELATIONS
INGRID MARLENE TAPIA – Management Secretary - INTERNATIONAL RELATIONS
RODRIGO MONSALVE – Manager - INTERNATIONAL RELATIONS
OFFICE OF – Office Parties Mailbox – HUMAN MANAGEMENT

Electronically Signed in Conformity with the Article 2º letter F and G of the Law 19.799
