# Supplementary material for: The KiVa antibullying program in primary schools in Chile, with and without the digital game component: study protocol for a randomized controlled trial
Source: Trials. 2017 Feb 20;18:75. doi: 10.1186/s13063-017-1810-1 (PMC5319041; doi:10.1186/s13063-017-1810-1)
Supplement: Additional file 4: — Funding Document from the Academy of Finland. (PDF 48.3 KB) [file 13063_2017_1810_MOESM4_ESM.pdf]

Academy of Finland

Funding decision

**Name**	Salmivalli, Christina
**Organisation**	TY
**Project title**	KiVa anti-bullying program in Chile: Evaluation of effectiveness with and without the digital game component
**Decision No.**	294199
**Decision date**	16.11.2015
**Funding period**	01.01.2016 - 31.12.2018
**Funding**	120 000

Project description

In Chile, there is an urgent need for evidence-based prevention of bullying. Despite some government-supported initiatives to help schools deal with bullying, no randomized controlled trials (RCTs) testing the effectiveness of existing anti-bullying programs have been done so far. The aim of the proposed project is to develop a culturally appropriate version of the KiVa antibullying program and to test its effectiveness in low-income primary schools in Santiago, Chile. Furthermore, it will be tested whether the digital learning environment, an anti-bullying computer game included in the Kiva program, adds to the effectiveness of the program. A randomized controlled trial will be conducted with three groups of schools: 1) the ones receiving the full KiVa program, 2) the ones receiving the KiVa program without the computer game, and 3) control schools.
