# Supplementary material for: The KiVa antibullying program in primary schools in Chile, with and without the digital game component: study protocol for a randomized controlled trial
Source: Trials. 2017 Feb 20;18:75. doi: 10.1186/s13063-017-1810-1 (PMC5319041; doi:10.1186/s13063-017-1810-1)
Supplement: Additional file 5: — Original Ethical Approval in Spanish. (PDF 63.3 KB) [file 13063_2017_1810_MOESM5_ESM.pdf]

Universidad de
los Andes

Dictamen

1. El Comité de Ética de la Universidad de los Andes, conformado por conformado por su Presidente (s), Carmen Sofía Brenes, profesora de Poética y Escritura de Guion; su secretario, Álvaro Pezoa, profesor de Ética Empresarial de la Escuela de Negocios, y sus integrantes Joaquín García-Huidobro, profesor de Ética y Pablo Zegers, profesor de Ingeniería Industrial, todos académicos de la Universidad, ha examinado el proyecto de investigación "KiVa anti-bullying program in Chile: Evaluation of effectiveness with and without the digital game component", del profesor Jorge Gaete (Universidad de los Andes), financiado por el Estado de Chile a través de la Comisión Nacional de Investigación Científica y Tecnológica (CONICYT) y por el Estado de Finlandia, a través de la Academia de Ciencias de Finlandia, con el objetivo de clarificar si contiene aspectos que merecen un examen desde el punto de vista de la ética de la investigación.

2. Este proyecto tiene por objetivo estudiar la efectividad del programa de antibullying KiVa, desarrollado en Finlandia, en colegios de Chile. En una **primera etapa**, se realizará la adaptación y validación de los cuestionarios que permitirán evaluar el programa finlandés. En la **segunda etapa** del estudio, se implementará el programa anti-bullying KiVa y se evaluará su efectividad de la misma forma que fue evaluado en Finlandia. En el caso chileno, además, se evaluará la efectividad de este programa con y sin el uso de un juego digital proporcionado a los alumnos.

3. El proyecto levantará información en un grupo de colegios: a alumnos, padres/apoderados y profesores.

4. Tanto el asentimiento informado como el consentimiento informado que se utilizará garantizan que se respetará la dignidad y la intimidad de las personas que se entreviste.

5. Analizado el proyecto, los miembros del Comité no ven riesgos de vulneración de los derechos de las personas que participen en las entrevistas.

Carmen Sofía Brenes
Presidente (s)

Álvaro Pezoa
Secretario

Dado en Santiago, a 18 de enero del 2016
