# Supplementary material for: The KiVa antibullying program in primary schools in Chile, with and without the digital game component: study protocol for a randomized controlled trial
Source: Trials. 2017 Feb 20;18:75. doi: 10.1186/s13063-017-1810-1 (PMC5319041; doi:10.1186/s13063-017-1810-1)
Supplement: Additional file 6: — English translation of Ethical Approval. (PDF 195 KB) [file 13063_2017_1810_MOESM6_ESM.pdf]

Universidad de los Andes

Approval Statement

1. The Ethics Committee of Universidad de los Andes, consisting of its President (s), Carmen Sofía Brenes, Professor of Poetics and Script Writing, its secretary, Álvaro Pezoa, Professor of Business Ethics of the Business School, and its members Joaquín García Huidobro, Professor of Ethics and Pablo Zegers, Professor of Industrial Engineering, all academics of the University, has examined the research project "KiVa anti-bullying program in Chile: Evaluation of effectiveness with and without the digital game component", by professor Jorge Gaete (Universidad de los Andes), funded by the State of Chile through the National Commission of Scientific and Technological Research (Comisión Nacional de Investigación Científica y Tecnológica, CONICYT) and by the State of Finland, through the Academy of Science of Finland, in order to clarify if it contains aspects that should be examined from the research ethics point of view.

2. This project aims to study the effectiveness of anti-bullying KiVa program, developed in Finland, in schools in Chile. In a first stage, the questionnaires that will assess the Finnish program will be adapted and validated. In the second stage of the study, the program will be implemented, and its effectiveness will be assessed in the same way that in Finland. In the Chilean case, the effectiveness of this program will be evaluated with and without the digital component provided to students.

3. This project will collect information in a group of schools about: students, parents/guardians and teachers.

4. Both, the informed assent and the informed consent that will be used, ensure that the dignity and privacy will be respected in the individuals that will be interviewed.

5. Analysed the project, Committee members see no risk of infringement of the rights of the persons participating in the study.

Carmen Sofía Brenes
President (s)

Álvaro Pezoa
Secretary

Given in Santiago, January 18th, 2016.
